# Synthesis and Characterization of Gelatin-Based Crosslinkers for the Fabrication of Superabsorbent Hydrogels

**DOI:** 10.3390/ma10070826

**Published:** 2017-07-19

**Authors:** Penphitcha Amonpattaratkit, Sureerat Khunmanee, Dong Hyun Kim, Hansoo Park

**Affiliations:** 1 Department of Integrative Engineering, Chung-Ang University, Seoul 156756, Korea; p.amonpattaratkit@gmail.com (P.A.); sureeratkhunmanee@gmail.com (S.K.); 2 Korea Institute of Industrial Technology, Gyeonggi 15588, Korea; dhkim@kitech.re.kr

**Keywords:** gelatin, methacrylic anhydride, itaconic acid, swelling, biodegradation, superabsorbent hydrogels

## Abstract

In this work, crosslinkers were prepared by conjugating high- and low-molecular-weight gelatin with different mole ratios of itaconic acid (IA) with double bonds. Then, the gelatin-itaconic acid (gelatin-IA) crosslinkers were compared with the gelatin-methacrylate (gelatin-MA) crosslinkers. The molecular weights and structures of gelatin-MA and gelatin-IA were confirmed using gel permeation chromatography (GPC) and nuclear magnetic resonance (NMR). Additionally, the swelling ratio and biodegradation properties of the hydrogels using IA as starting monomers and gelatin-IA and gelatin-MA as crosslinkers were investigated. Both hydrogels prepared with high and low molecular weights of gelatin-IA showed higher swelling ratios than those prepared with the gelatin-MA. The results also showed that absorbent hydrogels with different biodegradabilities and swelling ratios could be prepared by changing the ratio of the gelatin-based crosslinkers.

## 1. Introduction

Hydrogels are crosslinked networks of hydrophilic polymers that can swell in water and gain much higher masses. The physical and biochemical properties of hydrogels are largely dependent on their compositions, polymerization methods, and crosslinking density [1,2]. Moreover, superabsorbent hydrogels have been widely applied in many fields such as agriculture, drug-delivery systems, and hygienic products. Recently, natural (e.g., starch, cellulose, and gelatin) hydrogels have attracted much interest for alleviating environmental pollution [1]. Among them, gelatin derived from denatured collagen has been widely used in the food industry as an emulsifier, gelling and water retention agent, and in drug delivery because of its non-toxicity, storage stability, and cost-effectiveness [3,4,5].

Particularly, gelatin methacrylate (gelatin-MA), formed by incorporating methacrylate groups into the amine-containing side group of gelatin, has been investigated as a useful crosslinker for the preparation of network materials forming hydrogel systems in biomedical applications due to their biocompatibilities and natural cell binding [6,7,8,9]. Nichol and co-workers [7] studied surface and 3D cell binding, cell elongation, and migration properties of gelatin-MA. A photocrosslinkable interpenetration polymer network (IPN) hydrogel based on gelatin-MA and silk fibroin (SF) was fabricated by Xiao et al. [6] via sequential polymerization. Lai et al. prepared gelatin-MA-based hydrogels by adding carboxybetaine methacrylate (CBMA), leading to slower degradation, controlled drug release rate, and low toxicity of gelatin-MA/CBMA [10]. Moreover, gelatin-MA hydrogels have additional advantageous properties such as low cost, ease of production, and predictable degradation [6,7,10,11,12]. 

Recently, further improvements have been made in gelatin-based crosslinkers producing soft and flexible polymeric materials that can absorb a large amount of saline, water, or other solutions [13]. Itaconic acid (IA) is highly water-soluble due to its carboxylic groups, which may increase the hydrophilic characteristics of the polymer chains into which it is incorporated [14]. The preparation of natural polymers with IA-based hydrogels such as starch-*g*-acrylamide/itaconic acid, gum-poly(itaconic acid), and gelatin-*g*-poly(acrylic acid-*co*-itaconic acid)-based superabsorbent hydrogels have been synthesized [13,14,15]. However, there have been no systematic studies on the effect of the concentration of IA as well as the molecular weight (high (90,000 Da) and low (4800 Da)) of gelatin on a gelatin-based crosslinker (gelatin-IA) for hydrogel formation. In this study, crosslinkers based on high- and low-molecular-weight gelatin were synthesized with various IA ratios and compared with the gelatin-MA crosslinker. The effects of the molecular weight of gelatin and the ratio of MA to IA on the hydrogel characteristics were also investigated.

## 2. Results and Discussion

### 2.1. Characterization of High-Molecular-Weight Gelatin-MA (HGM) and Gelatin-IA (HGI) Crosslinkers and Hydrogels

**Figure 1:**
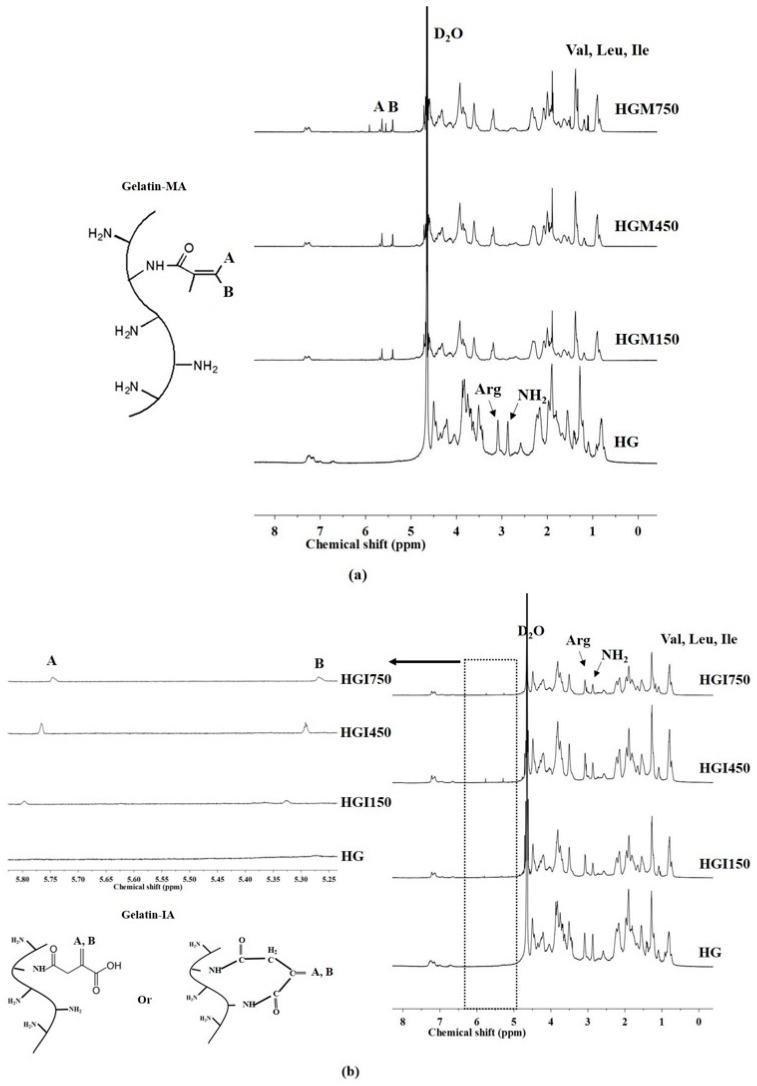
^1^H-NMR spectra of (**a**) High-Molecular-Weight Gelatin-MA (HGM) and (**b**) High-Molecular-Weight Gelatin-IA (HGI) crosslinkers.

The effects of high-molecular-weight gelatin-MA (HGM) and gelatin-IA (HGI) crosslinkers were investigated (Table 1). The formation of gelatin-based crosslinkers was confirmed by ^1^H-NMR. Figure 1 shows the ^1^H-NMR spectra of HGM (Figure 1a) and HGI (Figure 1b). New signals appeared between 5.2 and 6.0 ppm (A and B) in the spectra of HGM and HGI, indicating the conversion of additional functional groups in the gelatin molecules, demonstrating the successful conjugation of methacrylate and itaconic acid groups to the gelatin molecules. In addition, the disappearance or decrease of the intensity of the lysine methylene (NH_2_) signal at 2.9 ppm in HGM and HGI was observed after all the lysine residues were reacted, which agreed with the observations of Lai and coworkers [10]. These results suggest that gelatin was successfully modified at the molecular level by the conversion of functional groups [16]. 

**Figure 2:**
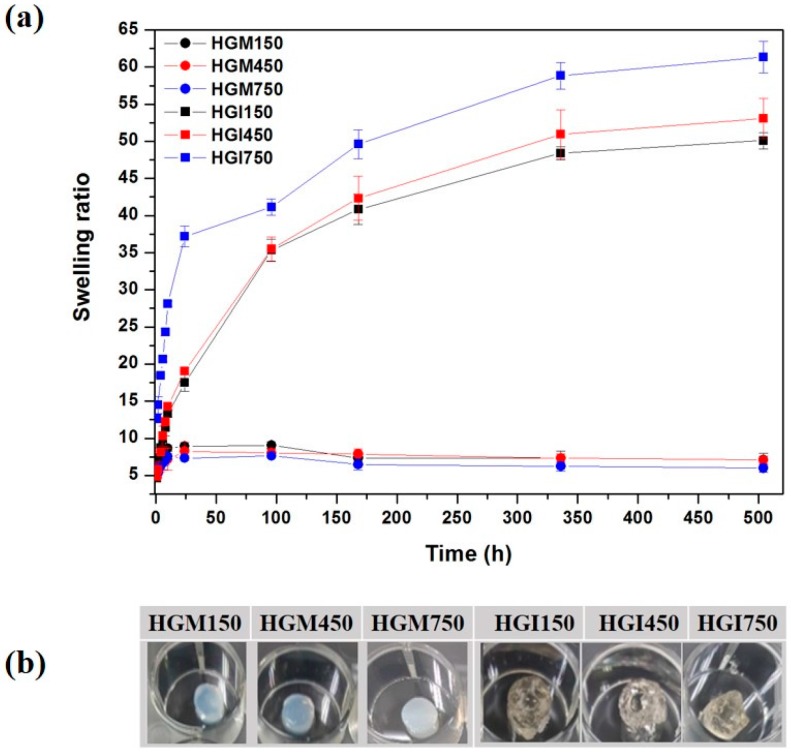
Swelling ratio (**a**) and photographs (**b**) of 9 w/v % of HGM and HGI hydrogels.

We confirmed the gel formation and shape of the HGM and HGI hydrogels, as shown in Figure 2. The swelling ratio of 9 w/v % HGM and HGI hydrogels demonstrated that HGM hydrogels did not show significant differences depending on the amount of MA incorporated. However, the swelling ratio of HGI hydrogels increased with the amount of IA. We believe that high degrees of grafting would result in a possible increase in the capacity of water absorption, since itaconic acid is a diprotic acid. The highest swelling ratio (38 g/g) was obtained at 750 mM of gelatin-IA. It was also observed that the swelling ratio of HGI hydrogels increased with longer periods of time and reached up to 60 g/g. This may be due to the large amount water diffused into the HGI hydrogels, leading to fast degradation followed by the enhancement of the swelling ratio [17]. 

**Figure 3:**
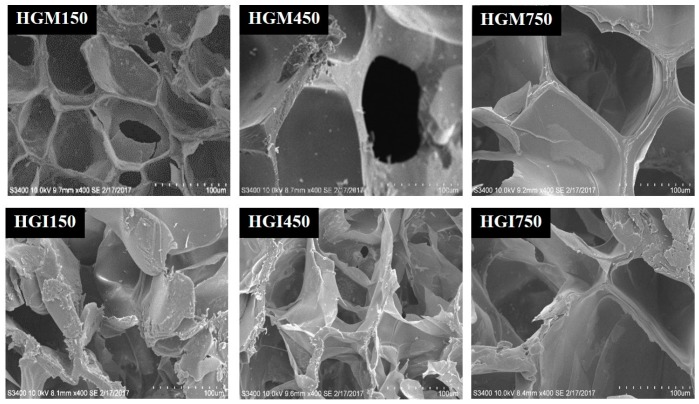
Scanning electron microscopy (SEM) photograph of 9 w/v % of HGM and HGI hydrogels.

The scanning electron microscopy (SEM) results depicted in Figure 3 show that both HGM and HGI hydrogels had interconnected pores. Capillary channels were also observed, which may enable water molecules to enter into the network. This observation is in agreement with that of Hosseinzadeh and coworkers, showing similar hydrogel structures [13]. Interestingly, while the composition of the 9 w/v % of gelatin-MA did not have a great effect on the microstructure of the hydrogels, the composition of gelatin-IA affected their microstructures, correlating directly with the swelling behaviors. It is believed that these pores are the regions of water permeation and interaction of external moieties with the hydrophilic groups of the hydrogels [12,18].

**Figure 4:**
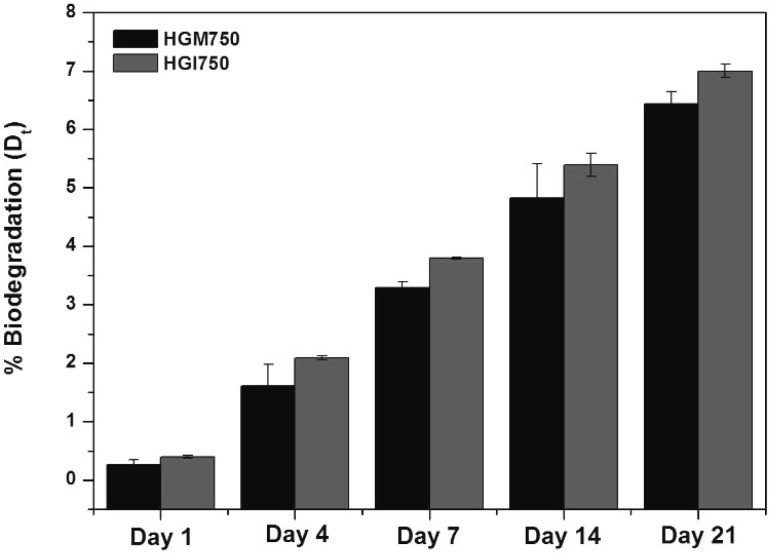
Percentage of biodegradation of 9 w/v % HGM750 and HGI750 hydrogels.

In this work, the percentage of biodegradation (D_t_) of HGI750 (which had the highest swelling ratio) and HGM750 (which had the lowest swelling ratio) hydrogels was measured by the continuous biochemical oxygen demand (BOD) test, as shown in Figure 4. The results show that the biodegradation of HGM750 hydrogel was slightly slower than that of the HGI750 hydrogel. This indicates that a higher swelling ratio and porosity increased water absorption, which may be responsible for the increase in the degradation of the samples [19]. However, the difference was not as large as we expected considering the dramatic difference in their swelling ratios. We believe that this could be due to the setup of the biodegradation test. In this experiment, biodegradation was measured by the oxygen consumption of microorganisms, and the water absorption might not affect this degradation rate since the gelatin is extremely biodegradable by microbial enzymes.

### 2.2. Characterization of High-Molecular-Weight Gelatin-IA (HGI) and Low-Molecular-Weight Gelatin-IA (LGI) Crosslinkers and Hydrogels

**Figure 5:**
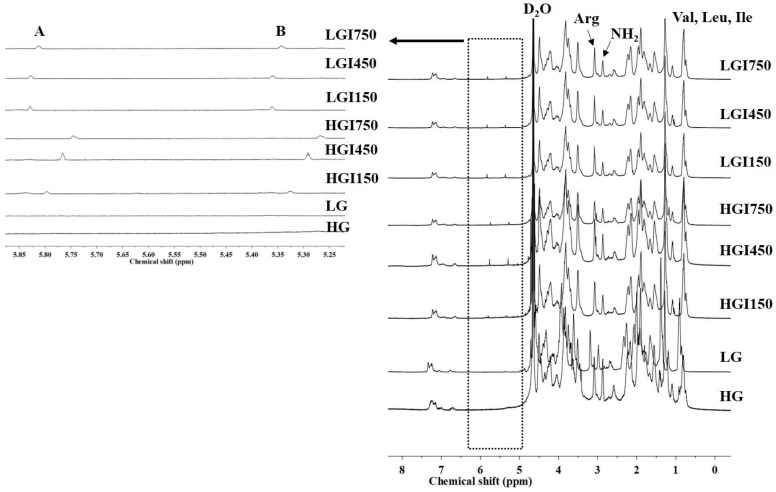
^1^H-NMR spectra of high-molecular-weight gelatin (HG), low-molecular-weight gelatin (LG), HGI, and LGI crosslinker.

Figure 5 shows the ^1^H-NMR spectra of high- and low-molecular-weight gelatin, HGI and LGI. Signals appeared in the spectra of the gelatin derivatives at 5.2 ppm ≤ δ ≤ 6.0 ppm, which confirms the conjugation of gelatin with IA, as discussed previously. 

**Figure 6:**
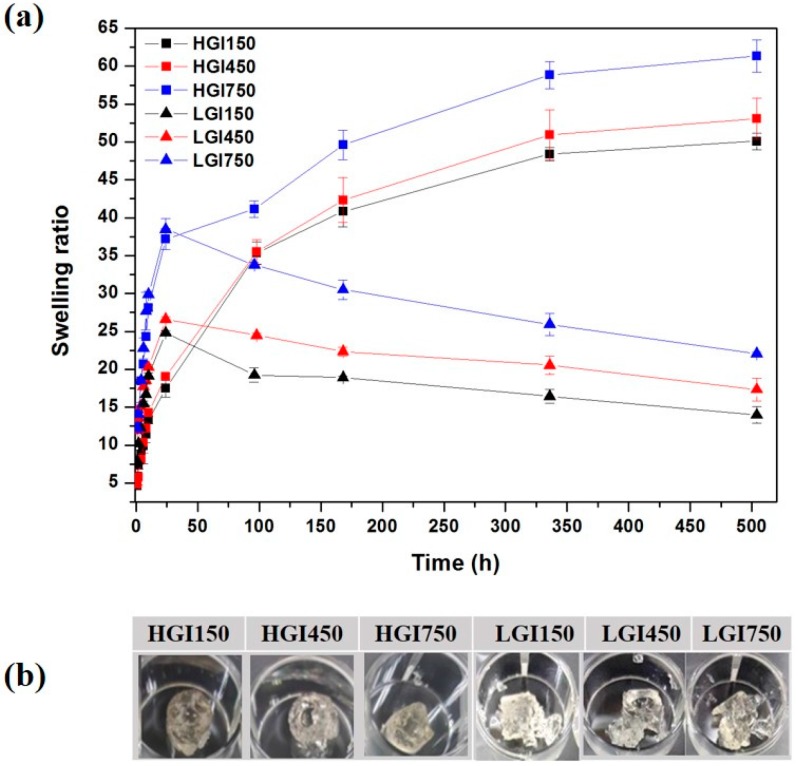
Swelling ratios (**a**) and photographs (**b**) of 9 w/v % HGI and LGI hydrogels.

As shown in Figure 6, the swelling ratio of the LGI hydrogel is slightly higher than that of the HGI hydrogel, which is in agreement with a previous report [20]. Collins and Birkinshaw investigated the effect of hyaluronic acid (HA) and molecular weight on the swelling ratios and degradation properties of hydrogels. It was found that low-molecular-weight HA hydrogel had higher swelling ratios and rapidly degraded after equilibrium water content was reached, compared with high-molecular-weight HA hydrogels. It was also observed that the swelling ratio of gelatin-IA hydrogels increased with increasing amount of IA in both LGI and HGI hydrogels. Interestingly, we observed a decrease in the swelling ratio of LGI hydrogels. This could be explained by the fact that the polymer chain in LGI hydrogels were dissolved and washed away after degradation, while the polymer chains still remained as part of the main chain for HGI hydrogels after degradation. 

**Figure 7:**
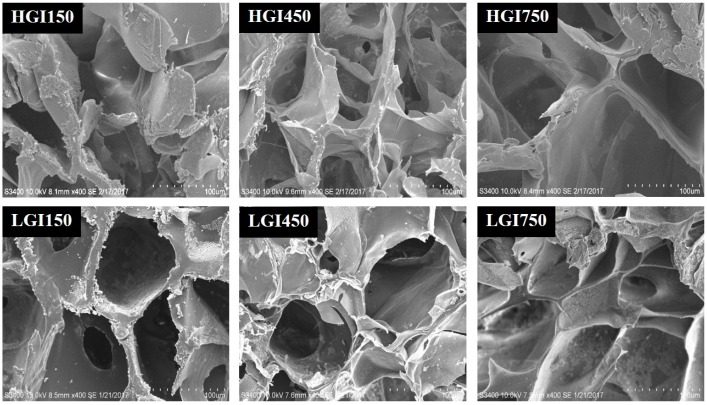
SEM micrograph of 9 w/v % HGI and LGI hydrogels.

In addition, the effect of various compositions of HGI and LGI hydrogels on their microstructures and swelling behaviors were studied, as shown in Figure 7. It was found that the microstructure of LGI hydrogels is similar to the microstructure of HGI and HGM hydrogels [13,18].

**Figure 8:**
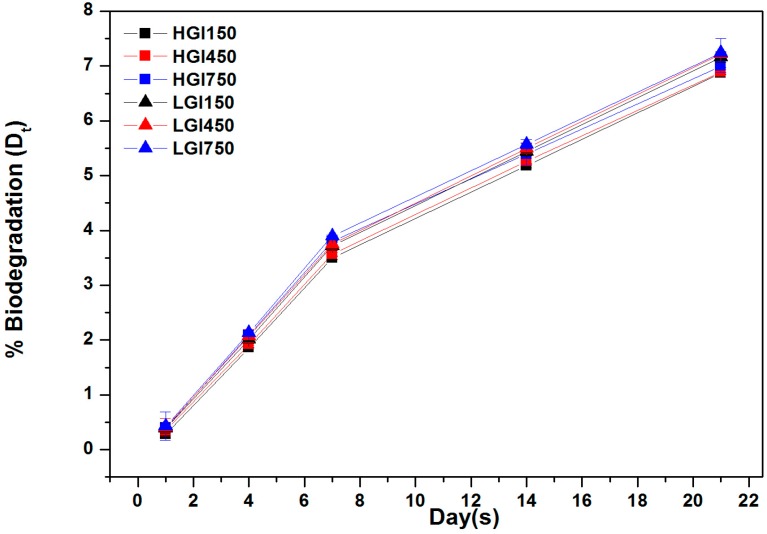
Percentage of biodegradation of 9 w/v % HGI and LGI hydrogels.

A comparison of the high and low molecular weights of HGI and LGI in terms of their biodegradation properties is shown in Figure 8. Biodegradation slightly increased with increasing amounts of itaconic acid. The degradation of the hydrogels was apparent as a loss of physical coherence and identity in the swelling medium [20]. In addition, the biodegradation of LGI was slightly higher than that of HGI. We also found only a small difference in the degradation rate between LGI and HGI hydrogels, which was consistent with previous experiments. After the formation of the gelatin-IA network, the molecular weight of gelatin had no significant effect on the microstructure and biodegradation of the gelatin-IA hydrogels, but it did affect the swelling ratio.

## 3. Materials and Methods

### 3.1. Fabrication of High-Molecular-Weight Gelatin-MA (HGM) and Gelatin-IA (HGI), and Low-Molecular-Weight Gelatin-IA (LGI) Crosslinkers

Gelatin (Type A, 300 bloom from porcine skin, ~90,000 Da), methacrylic anhydride (MA), itaconic acid (IA), *N*,*N*,*N*’,*N*’-tetramethylethylenediamine (NHS), initiator ammonium persulfate (APS), and terminator *N,N,N′,N′*-tetramethylethylenediamine (TEMED) were purchased from Sigma-Aldrich (St. Louis, MO, USA). 1-ethyl-3-(3-dimethylaminopropyl) carbodiimide hydrochloride (EDC) was purchased from Thermo Scientific (Rockford, USA). For gelatin hydrolysis, a 5 w/v % gelatin solution was prepared at room temperature. Its pH value was adjusted to pH 8 with 0.5 M NaOH. Afterwards, the solution was placed in a water bath maintained at 80 °C and stirred for 4.5 h. The mixture was dialyzed for 3 days against distilled water at room temperature. The molecular weight of the gelatin after hydrolysis (~4800 Da) was investigated by gel permeation chromatography (GPC).

High- and low-molecular-weight gelatins (HG and LG) were mixed into a Dulbecco’s phosphate buffered saline (DPBS; Hyclone) solution at 60 °C and stirred for 2 h until complete dissolution. MA was added dropwise, and IA was added with EDC and NHS for gelatin-MA and gelatin-IA preparation, respectively, under an Ar atmosphere with vigorous stirring. The different compositions used for the synthesis are shown in Table 1. After 5 h, additional DPBS was added at 60 °C for 15 min to terminate the reaction. The mixture was dialyzed for 1 week against distilled water at 40 °C (for gelatin-MA) and room temperature (for gelatin-IA) to remove salt and unreacted MA and IA. The solutions were stored at −80 °C until frozen and then lyophilized for 48 h to form a white porous foam.

### 3.2. Fabrication of HGM, HGI, and LGI Hydrogels

For hydrogel preparation, 9 w/v % of the dried gelatin-MA and gelatin-IA crosslinkers were mixed into DPBS at 37 °C in a water bath until fully dissolved to prepare gelatin-MA and gelatin-IA solutions. APS (initiator), IA (monomer), and TEMED (terminator) solutions (0.3 M) were also prepared with DPBS. Finally, the hydrogel was lyophilized for 48 h.

### 3.3. Characterization of HGM, HGI, and LGI Crosslinkers and Hydrogels

The functional groups of gelatin-MA and gelatin-IA were identified by nuclear magnetic resonance (^1^H-NMR, Varian, Palo Alto, CA, USA). ^1^H-NMR spectra were collected at 35 °C in deuterium oxide (Sigma-Aldrich) at a frequency of 600 MHz. The microstructures of gelatin-MA and gelatin-IA hydrogels were investigated by SEM. Dried hydrogel samples were coated with platinum and imaged in an SEM instrument (Hitachi S-3400N, Tokyo, Japan). For swelling measurements, gelatin-MA and gelatin-IA hydrogels were immersed in distilled water and allowed to soak for 1, 2, 4, 6, 8, 10, 24, 96, 168, 336, and 504 h at room temperature. The swelling ratio was calculated according to Equation (1):

Swelling ratio = W_s_ − W_d_/W_d_ (1)

where W_d_ and W_s_ are the weights of the dry and swollen hydrogels, respectively.

For the biodegradation test, the percentage biodegradation (D_t_) of high-molecular-weight gelatin-MA (HGM) and high- and low-molecular-weight gelatin-IA (HGI and LGI) hydrogels was investigated for 1, 4, 7, 14, and 21 days according to ISO 14851:1999. First, a microorganism solution was prepared by mixing solid solution diluted with standard media 5 v/v %. After that, air was supplied by an air pump and oxygen concentration was checked for saturation at ~8.3 mg/L by a dissolved oxygen meter. Each hydrogel, after freeze-drying (1 g), was mixed with 200 mL of microorganism solution in a BOD bottle. All sample bottles were kept in an incubator at 25 °C and spun at 100 rpm. After that the oxygen concentration of sample was checked every 1, 4, 7, 14, and 21 days. Moreover, after the measurement ay day 7, re-aerated solutions with saturated oxygen concentrations were added to the bottles. 

Percentage biodegradation (D_t_) was calculated as the ratio of specific biochemical oxygen demand (BODs) to the theoretical oxygen demand (ThOD), using Equation (2):

D_t_ = BODs/ThOD × 100 (2)

The ThOD (mg/mg) of a substance C_c_H_h_Cl_cl_N_n_S_s_P_p_Na_na_O_o_ of relative molecular mass M_r_ can be calculated using Equation (3):

ThOD = 16[2c + 0.5 (h-cl-3n) + 3s + 2.5p + 0.5na − o]/M_r_ (3)

The BODs were calculated using Equation (4):

BODs = U_r_ × O_c_ (4)

where U_r_ and O_c_ are the relative oxygen uptake and total oxygen capacity, respectively.

Determination of the U_r_ measured in the aqueous phase in each bottle was performed using Equation (5):

U_r_ = C_Br_ − C_t_/C_s_ (5)

where C_Br_, C_t_, and C_s_ are the dissolved-oxygen concentrations (mg/L) after incubation at time t in the blank bottles and each test bottle, and the saturation value for dissolved oxygen (8.3 mg/L), respectively. 

Determination of the O_c_ of a bottle from the maximum oxygen was performed using Equation (6):

Oc = (0.28 × V_g_) + (0.009 × V_l_) (6)

where V_g_ and V_l_ are the volume of gas and liquid in an incubation bottle (mL), respectively. 

## 4. Conclusions

Gelatin-MA- and gelatin-IA-based hydrogels were synthesized to study the effects of different mole ratios of gelatin:MA and gelatin:IA on the microstructure, swelling, crosslinking behaviors, and biodegradability of the hydrogels. The conjugation of gelatin with MA and IA was confirmed by ^1^H-NMR. The highest swelling ratio was found for the low-molecular-weight gelatin-IA hydrogel. The swelling ratio of the gelatin-IA hydrogels increased with increasing IA content. Furthermore, a porous structure was found in all the hydrogels. The most biodegradation was recorded for the LGI750 low-molecular-weight gelatin-IA hydrogel. 

## Figures and Tables

**Table 1:** Compositions with different mole ratios of methacrylic anhydride (MA) and itaconic acid (IA) and high and low molecular weights of gelatin.

Abbreviation	Molecular Weight of Gelatin (Da)	Amount of MA or IA (mM)	Gelatin:MA or IA (Mole Ratio)
HG	90,000	-	-
LG	4800	-	-
HGM150	90,000	150	1:540
HGM450	90,000	450	1:1620
HGM750	90,000	750	1:2700
HGI150	90,000	150	1:540
HGI450	90,000	450	1:1620
HGL750	90,000	750	1:2700
LGI150	4800	150	1:30
LGI450	4800	450	1:90
LGI750	4800	750	1:150
